# Morphological and Molecular Characterization of Some Egyptian Six-Rowed Barley (*Hordeum vulgare* L.)

**DOI:** 10.3390/plants10112527

**Published:** 2021-11-20

**Authors:** Azza H. Mohamed, Ahmad A. Omar, Ahmed M. Attya, Mohamed M. A. Elashtokhy, Ehab M. Zayed, Rehab M. Rizk

**Affiliations:** 1 Department of Agricultural Chemistry, College of Agriculture, Mansoura University, Mansoura 35516, Egypt; 2 Citrus Research and Education Center, University of Florida (IFAS), Lake Alfred, FL 33850, USA; 3 Biochemistry Department, Faculty of Agriculture, Zagazig University, Zagazig 44519, Egypt; 4 Barley Research Department, Field Crops Research Institute, Agricultural Research Center, Giza 12619, Egypt; ahmed_1099@hotmail.com; 5 Genetics Department, Faculty of Agriculture, Zagazig University, Zagazig 44511, Egypt; mmabdeltawab@zu.edu.eg; 6 Cell Study Research Department, Field Crops Research Institute, Agricultural Research Center, Giza 12619, Egypt; ehabzayed977@gmail.com; 7 Botany Department, Faculty of Science, Mansoura University, Mansoura 35516, Egypt; rehabrizk@mans.edu.eg

**Keywords:** plastid markers, DNA barcoding, ISSR markers, Egyptian barley, agro-morphological traits, cluster analysis, genetic variation, biplot

## Abstract

Barley production is essential in Egypt. In the present study, 15 different six-rowed Egyptian barley cultivars were studied. To differentiate between the different cultivars under study in terms of morphological characteristics and ISSR, molecular characterization reactions were carried out. Moreover, four cultivars (Giza 123, Giza 126, Giza 136, and Giza 138) were selected for further studies using scanning electron microscopy (SEM). Computational analysis of the DNA barcoding sequences of the two plastid markers *rbc*L and *mat*K was executed, and the results were deposited in the NCBI database. The morphological traits showed low statistical significance among the different cultivars under study via the data collected from two seasons, suggesting that the mean field performance of these Egyptian cultivars may be equal under these conditions. The results showed that the phylogenetic tree was divided into four groups, one of which contained the most closely related genotypes in the genetic distance, including Giza 124, Giza 130, Giza 138, Giza 136, and Giza 137, which converge in the indicative uses of farmers. The seed coat of the studied cultivars was “rugose”. The elevation folding of the rugose pattern ranged from 11 ± 1.73 µm (Giza 126) to 14.67 ± 2.43 µm (Giza 123), suggesting variation in seed quality and its uses in feed and the food industry. According to the similarity matrix of ISSR analysis, the highest similarity value (93%) was recorded between Giza 133 and Giza 132, as well as between Giza 2000 and Giza 126. On the other hand, the lowest similarity value (80%) was recorded between Giza 130 and (Giza 133 and Giza 132), indicating that these cultivars were distantly related. Polymorphism information content (PIC) ranged from 0.26 for the primer ISSR UBC 835 to 0.37 for the primers ISSR UBC 814 and ISSR UBC 840. The current study showed that the *mat*K gene is more mutable than the *rbc*L gene among the tested cultivars.

## 1. Introduction

Barley (*Hordeum vulgare* L.) is one of the main and oldest cereal crops on Earth. Worldwide, its grain production is ranked fourth after maize, rice, and wheat [1]. Barley is generally considered a poor man’s crop because it is easy to cultivate, with few requirements, and has a high capacity for adaptation to harsh environments. Some literature estimates the age of barley at 11,000 years [2]. However, six-rowed barley did not arise until after 6000 BC [3]. Archaeological evidence has dated barley cultivation to 5000–6000 BC in Egypt [4,5,6,7]. Barley products, especially bread and beer, comprised a complete diet in ancient Egypt. Based on the health benefits of barley, as well as the need for agricultural development and reducing wheat imports, Egypt is currently examining the return of barley to the bread-making industry as a 30% ingredient. The global production volume of barley reached 142.37 million metric tons in the 2017/2018 crop year [1]. Furthermore, it is expected that barley production will decrease to 140.6 million metric tons in the next crop year [8]. Egypt’s barley production has fluctuated substantially in recent years as it increased over the past two decades, ending at 108,000 tons in 2019 [1]. With the new policies for the sustainable development plan and reclaimed land expansion, the area and productivity are expected to increase.

There are approximately 38 Egyptian barley cultivars—two- and six-rowed—but the six-rowed barleys are the most famous and widely used in Egypt. Field evaluations have shown many differences between genotypes [9,10]. Furthermore, [11] found that all studied traits showed significant differences between genotypes, environments, and interactions. Moreover, Sharma, et al. [12] used Euclidean distances based on non-hierarchical cluster analysis to categorize total accessions into diverse clusters, and to determine and select accessions with decent yield and performance for other ancillary traits. The candidate breeding lines can be used in hybridization for barley improvement programs.

Molecular markers are an essential tool used to directly detect the differences between and within genetic materials at the DNA level; they provide a robust estimate of genetic similarity that is not often obtained using morphological data alone [13]. A comparison can also be made to determine the genetic distance between the Egyptian cultivars based on field characteristics and molecular parameters. The inter simple sequence repeats (ISSRs) technique has been successfully applied to many crop species [14,15,16]. ISSRs demonstrate the specificity of microsatellite markers, and require no specific sequence information for primer synthesis, using the advantage of random markers [17]; thus, they have been widely used for cultivar identification in different crops [18,19,20]. Moreover, Guasmi et al. [17] found that ISSR primers exhibited variations in the percentage of polymorphism, resolving power (Rp), and band informativeness (Ib); the rate of polymorphism was 66.67%, the Rp ranged from 0.74 to 1.16, and the average Ib ranged from 0.24 to 0.39, suggesting that ISSRs are robust molecular markers that can distinguish between Egyptian cultivars. On the other hand, according to Drine, et al. [21], ISSRs and random amplified polymorphic DNA (RAPD) markers identified 72.2% and 61% of polymorphic bands, respectively. Several parameters were used to compare the relative efficiency of these marker systems, including the effective multiplex ratio (EMR), marker index (MI), and polymorphic information content (PIC); the ISSR system showed higher values for all of the parameters examined. Moreover, Wang, et al. [22] used 10 ISSR primers to investigate the variation between Tibetan and Middle Eastern barley genotypes; the Tibetan genotypes contained 91 allelic variants, of which 79 were polymorphic (86.81%), while the Middle Eastern genotypes contained 82 allelic variants, of which 66 were polymorphic (80.49%). These results suggest that ISSRs are robust molecular markers that can be employed to distinguish Egyptian cultivars.

The morphology of barley grains observed via scanning electron microscopy (SEM) showed starch granules smaller than the standard ones; they appeared in abnormal shapes with a conspicuous peripheral groove and sunken cheeks [23,24]. When SEM was used to study the detailed structure, it proved that starch was degraded by both pitting and surface erosion [25]. The apparent shape of the seed under the electron microscope indicates the quality of its industrial and agricultural importance.

DNA barcoding is a genetic identification technology that uses a genetic region of short DNA sequences, called the DNA barcode [26]; this can be reliably characterized by similar morphological characteristics and chemical compositions [27]; it has two main objectives: identifying organisms, where an unknown sequence matches a known species sequence, and exploring species that are similar in terms of habitat delimitation and description of species [28]. A short DNA sequence obtained from established target regions of the chloroplast genome can be used to classify genera and/or species of plants with respect to orthologous databases, compared to conventional PCR-based markers [29]. DNA barcoding has been proposed as an essential tool for resolving the significant gaps in our current understanding of biodiversity. Furthermore, Barley and Thomson [30] demonstrated that the success of DNA barcoding varies broadly across DNA substitution models, and has a substantial influence on the number of operational taxonomic identified units. Moreover, using recent advances in combinatorial pooling and next-generation sequencing, Lonardi, et al. [31] proposed a new sequencing approach that addresses the challenge of de novo selective genome sequencing in a highly efficient manner. Barcodes can be employed to explain the relationships between Egyptian cultivars, and their relation to sequences within the database.

The main objective of this study was to measure and characterize the differences between the most economically important Egyptian barley cultivars, especially in making bread. The study investigated 15 Egyptian six-rowed cultivars at the field level, the molecular level, via scanning electron microscopic examination, and via DNA barcoding. The results obtained from the present study will potentially enhance breeding programs and lead to the development of new adaptive or high-yield barley cultivars with specific improved traits.

## 2. Results and Discussion

### 2.1. Field Experimental

#### 2.1.1. Growing across Two Seasons

**Figure 1:**
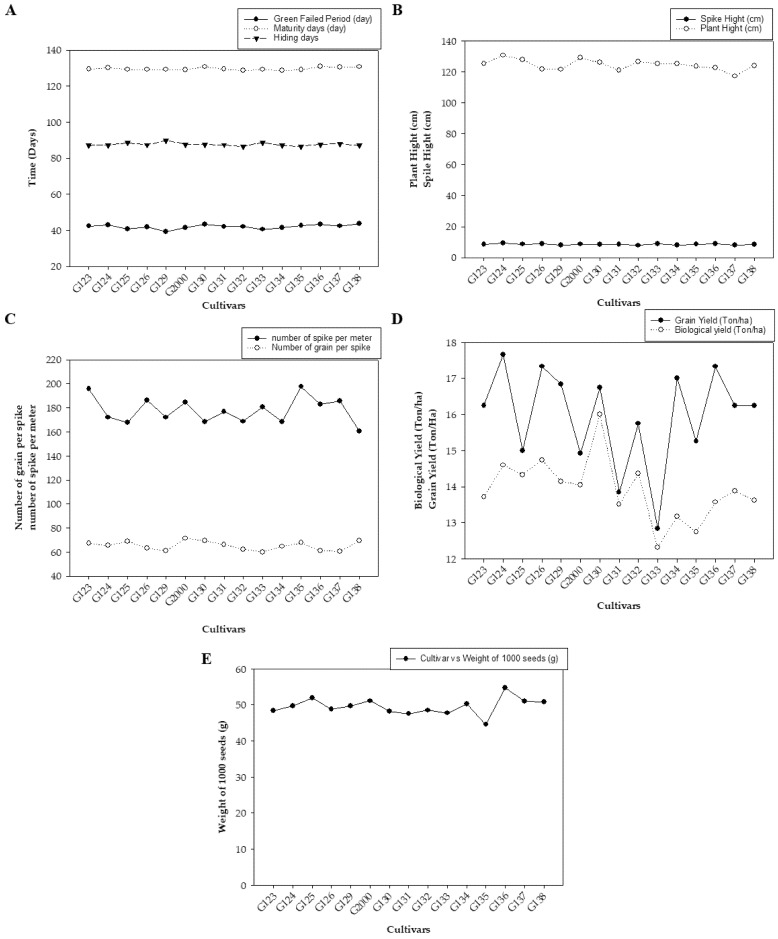
The averages of morphological traits in 15 barley genotypes grown in two seasons—2017/2018 and 2018/2019: (**A**) grain filing period (day), maturity day (day), and hiding day (day); (**B**) spike height (cm) and plant height (cm); (**C**) number of spikes per square meter and number of grains per spike; (**D**) biological yield (ton/ha) and grain yield (ton/ha); and (**E**) weight of 1000 grains (g).

Figure 1 represents the average values of the field data during the two growing seasons, including grain filing period (day), maturity day (day), and hiding day (day) (Figure 1A); spike height (cm) and plant height (cm) (Figure 1B); the number of spikes per square meter and number of grains per spike (average of 10 spikes per square meter) (Figure 1C); biological yield (ton/ha) and grain yield (ton/ha) (Figure 1D); and weight of 1000 grains (g) (Figure 1E). The average mean values showed low statistical significance among the examined cultivars. There were no significant differences based on the least significant difference (LSD) for any of the studied traits except for biological weight, which showed a significant difference in values between the different genotypes (LSD = 1.78).

Figure 1 shows differences between genotypes in all studied traits, which were divided into three parts according to the convergence of the numerical values of the traits. There were indications of early cultivars being equal through the periods of maturity and seed fullness, and low statistical significance among them (Figure 1A). Plant height indicated vegetative solid growth (Figure 1B), which is sufficient for animal feed. These results are consistent with those of Amer, et al. [32], who found that the average yield of the new cultivar Giza 137 was 16.7 4.95 Ton/ha, while that of Giza 138 was 5.07 Ton/ha. These yields significantly exceeded the national checks Giza 123 and Giza 132 (3.88 Ton/ha). Giza 137 significantly out-yielded Giza 123 and Giza 132, by ~22.4 and ~20.7%, respectively. Furthermore, Giza 138 significantly exceeded the average of national checks Giza 123 (by ~25.6%) and Giza 132 (by ~23.9%).

On the other hand, Noaman, et al. [33] found that biological weight characterized new genotypes. Furthermore, Mariey, et al. [34] considered the Egyptian barley genotypes Giza 123, Giza 131, and Giza 136 to be salt tolerant. It is worthy of note that this study was performed under the conditions and climate of Giza Governorate, Egypt. Furthermore, the behavior of the varieties differs when studied under different environmental conditions—such as in the Sinai Peninsula or on the northern coast—even though they have the same genetic background; the same can be said of their other behavior under harsh conditions.

#### 2.1.2. Genetic Distance Dendrogram between Genotypes Based on Field Traits

**Figure 2:**
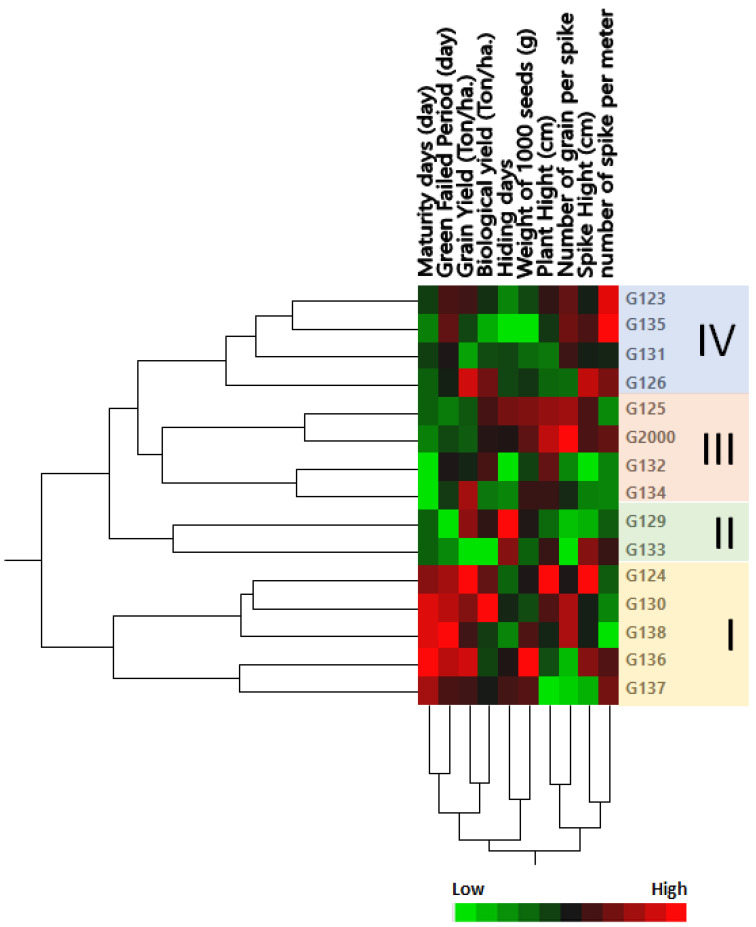
Cluster analysis and heatmap based on agro-morphological traits of 15 Egyptian six-rowed barley cultivars. The heatmap was constructed using JMP^®^, Version 15 (SAS Institute Inc., Cary, NC, USA, 1989–2019).

The genetic tree of the genotypes was divided into four groups: Group I contained the most closely related genotypes in terms of genetic distance, including Giza 124, Giza 130, Giza 138, Giza 136, and Giza 1137 (Figure 2); members of this group were characterized by a high maturity day and high grain filing period (days), along with grain yield (Ton/ha) and biological yield (Ton/ha). Group II contained the two cultivars Giza 129 and Giza 133; this group could be described by a low number of grains per spike and high hiding days. Group III consisted of Giza 125 and Giza 2000 on one side of the group, and Giza 132 and Giza 134 on the other side (Figure 2); members of this group were characterized by high plant height and low-to-moderate maturity days. Group IV consisted of Giza 123, Giza 135, Giza 131, and Giza 126; this group could be characterized by height, a moderate number of spikes per square meter, spike height (cm), and the number of grains per spike, along with low-to-moderate weight of 1000 grain (g), hiding days, biological yield (Ton/ha), and maturity days (Figure 2). These results were consistent with the findings of Mariey and Khedr [35]. Moreover, based on their 10 agro-morphological traits, Mareiy, et al. [36] explored biplot and cluster analysis using Euclidean distance matrices and average linkage. According to PCA, all 15 genotypes fell into 4 groups. Cultivars in Group A tend to have higher yields, so they may be considered to be tolerant (Giza 16 and Giza 18). Nevertheless, Giza 124, Giza 132, and Giza 134 are among the cultivars in group D that produce lower grain yields. The characteristics of biological weight and biological yield are used to assess the production of grain in relation to the rest of the plant components, which are used as animal feed in the form of straw. Indeed, increasing the seed yield and decreasing the biological crop is beneficial to grain production, which is the goal of growing barley for nutrition and intensive production.

### 2.2. Scanning Electron Microscopy (SEM)

**Figure 3:**
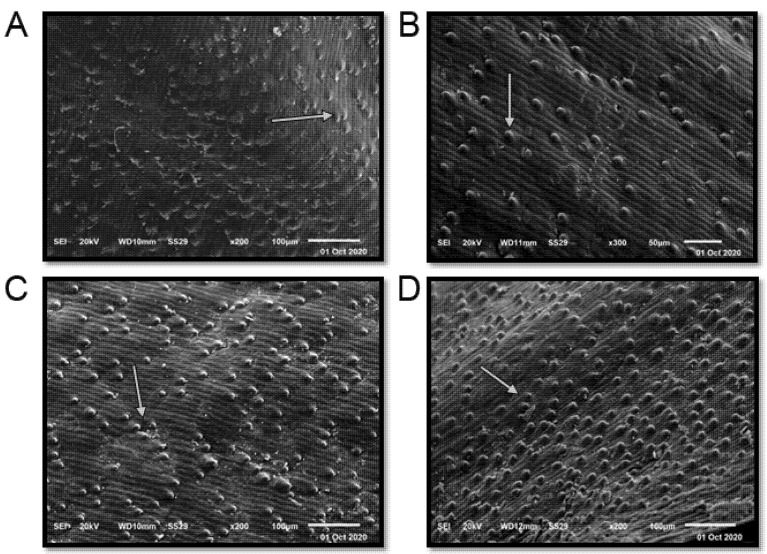
Scanning electron microscope (SEM) images of four Egyptian six-rowed barley (*Hordeum vulgare* L.) cultivars: (**A**) Giza 123, (**B**) Giza 126, (**C**) Giza 136, and (**D**) Giza 138. Scale bar = 100 µm. White arrows indicate the quality and shape of the rugose.

The term seed coat of barley caryopsis includes tissues from three separated organs: the pericarp, the testa, and the semipermeable membrane. Several unique compounds are synthesized in the seed coat, serving the plant’s defense and control of its development in different ways. Additionally, many of these compounds are sources of industrial products and components for human consumption or animal feed [37]. The seed coat of the studied cultivars is “rugose” (Figure 3 and Table 1). The elevation folding of the rugose pattern ranges from 11 ± 1.73 µm (Giza 126) to 14.67 ± 2.43 µm (Giza 123). The extension of the rugose pattern (length) ranges from 16.00 ± 2.61 µm (Giza 126) to 18.67 ± 3.13 µm (Giza 136). The frequency pattern in 100 µm^2^ ranges from 4.67 ± 0.51 (Giza 126) to 12.17 ± 1.69 (Giza 138). Thus, these cultivars could be promising for different purposes in service of contemporary Egyptian interests.

### 2.3. Molecular Characterization and Genetic Relationships as Revealed by ISSR Markers

**Figure 4:**
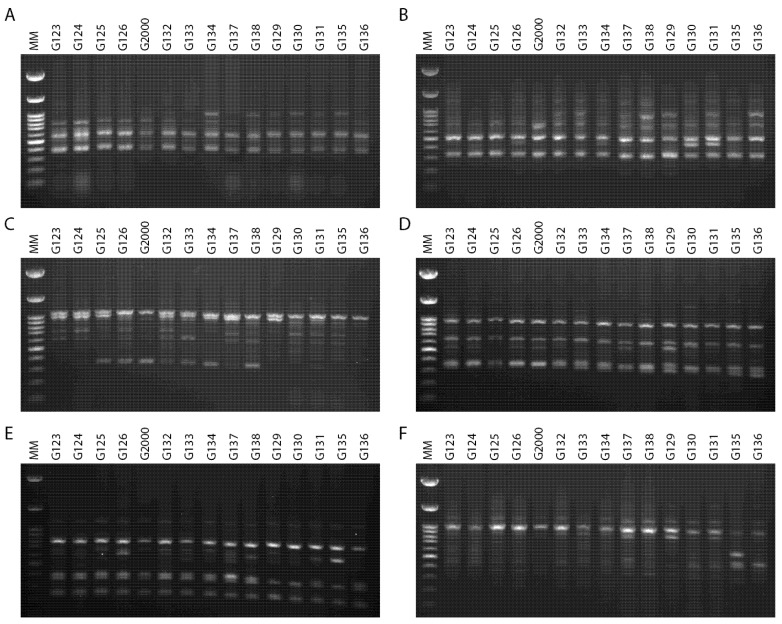
ISSR–PCR product profiles of 15 investigated samples of *Hordeum vulgare L*: (**A**) primer UBC 814, (**B**) primer UBC 826, (**C**) primer UBC 840, (**D**) primer UBC 808, (**E**) primer 807, and (**F**) primer 851. M: molecular size marker (100 bp).

The ability to effectively utilize genetic variability available to breeders is dependent upon an understanding of population diversity [38,39]. Thus, the primary benefit of cultivar differentiation at the molecular level is to explain with some accuracy the relationships between cultivars, in order to reduce selection costs within breeding programs and provide future breeders with molecular insights. The inter simple sequence repeats (ISSRs) fingerprinting profiles generated by 4 out of the 15 primers used in the present study, targeting 15 Egyptian six-rowed cultivars of barley, are displayed in Figure 4. The polymorphism generated by the 15 ISSR primers is summarized in Table 2. The 15 ISSR primers used in the present study produced a total number of bands (TNB) of 126, and 62 of those were polymorphic with uniqueness (PWU), with a polymorphism percentage (P%) of 50.07%. The TNB ranged from 5 for the ISSR UBC 844A and ISSR UBC 901 primers, to 14 for the ISSR UBC 835 primer. The number of PWU bands also varied, from two in ISSRs UBC 825 and UBC 901, to seven bands in the ISSR 857 and ISSR UBC 835 primers. The average number of PWU bands was 4.13 per primer (Table 2). The polymorphism information content (PIC) values varied between the ISSR primers. PIC ranged from 0.26 for the primer ISSR UBC 835 to 0.37 for the primers ISSR UBC 814 and ISSR UBC 840. Remarkably, some ISSR primers revealed distinct discrimination of 22–80% polymorphism, including ISSR UBC 825 and ISSR UBC 844A (Table 2). The ISSR primer UBC 844A recorded the lowest effective multiplex ratio (EMR) (6.20) and lowest marker index (MI) (0.02), whereas ISSR UBC 814 scored the highest in terms of PIC, resolving power (RP), and MI values (0.37, 12.67, and 0.05, respectively). Furthermore, ISSR UBC 835 scored the lowest values for PIC and MI (0.02 and 0.26, respectively) and the highest value in EMR (12.07).

Genetic diversity in some six-rowed barley cultivars grown in Egypt was assessed using ISSR markers. The 15 ISSR primers produced 97 markers that were utilized to investigate the genetic diversity among the studied cultivars. A polymorphism percentage of 50.07%, with an average of 4.13 markers per primer, was found among the studied cultivars (Table 2). However, this number ranges from two for ISSR UBC 825 and ISSR 857, to seven for ISSR UBC 835. The ISSR primers produced single and unique bands, and four molecular primers had these bands (ISSR UBC 814, ISSR UBC 827, ISSR 807, and ISSR 851). The use of ISSR markers for fingerprinting previously resulted in high polymorphism between species, and reflected intraspecific variations within species [14,15,40]. In addition to the high level of polymorphism observed in the current study by ISSR, this may imply high insertional activity in the genome of the tested barley cultivars [21,41,42].

**Figure 5:**
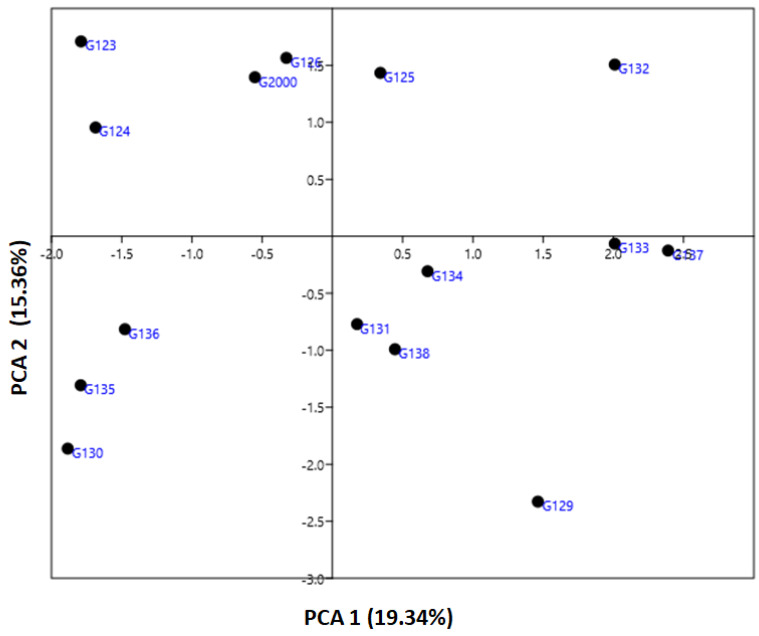
An illustration of the genetic diversity expressed in 15 Egyptian six-rowed barley cultivars, according to a principal component analysis (PCA) based on polymorphism of ISSR markers, using PAST software.

The genetic diversity parameter data revealed by ISSR markers were utilized to calculate the genetic diversity of the studied cultivars by using multivariate clustering, PCA, and heatmap analyses. In a PCA scatterplot, the ISSR markers reflect the robustness of the markers in categorizing the investigated cultivars. PCA analysis indicated that the four six-rowed Egyptian barley cultivars Giza 126, Giza 2000, Giza 125, and Giza 132 were distinct from the other cultivars (Figure 5). Neighboring affinity was also apparent between the Giza 135, Giza 136, and Giza 130 cultivars (Figure 5). Conversely, the rest of the cultivars—Giza 129, Giza 138, Giza 131, Giza 133, Giza 134, and Giza 137—were scattered at some distance from one another. The cultivars Giza 126 and Giza 2000 were the best foragers, as designated by cluster analysis (Figure 5), which also indicated a significant distance between Giza 123 and Giza 124 (Figure 5), and between Giza 132 and Giza 135, Giza 136, and Giza 130 (Figure 5). The differentiation of the studied cultivars in terms of years of release and pedigree may be due to previous alterations in production conditions. There is a possibility that these morphological characteristics can increase or decrease genetic variation between cultivars. Data from ISSR markers analyzed in this study might be explained by the instability of TNB insertion events, cultivar production, and behavior under environmental conditions [17,35]. There may be a correlation between the high degree of polymorphism observed in ISSR markers and genotype diversity [16,21,43]. Although there were differences between the dendrograms based on field characteristics, and in PCA results based on the molecular parameters, both sorted the cultivars into four groups closer to their uses in Egypt.

**Figure 6:**
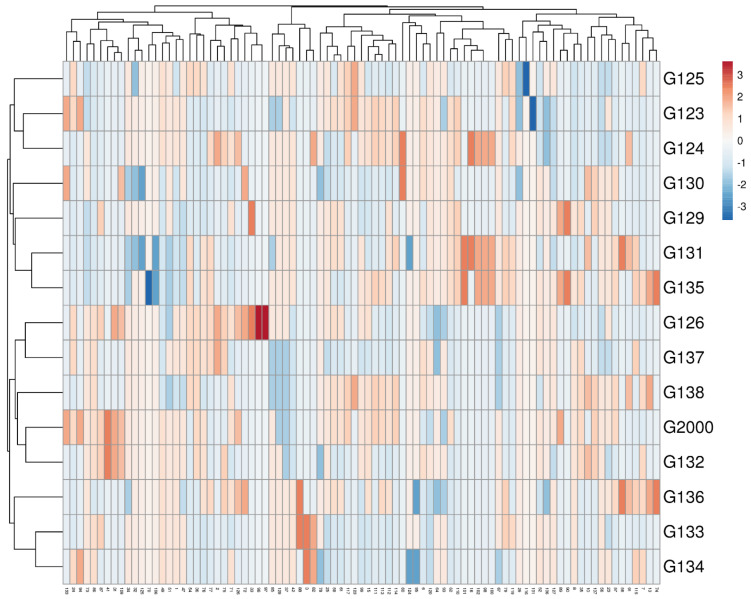
Multivariate heatmap illustrating the genetic diversity of 15 Egyptian six-rowed barley cultivars, based on the 15 ISSR primers for using the module of a heatmap of ClustVis—an online tool for clustering and visualizing of multivariate data [41].

Multivariate compound similarity analysis is usually utilized to show more information about the genetic variance of plant breeds, which is detailed in heatmaps [40]. The multivariate compound similarities were presented as a heatmap constructed using R software. As indicated by the columns, 15 Egyptian barley cultivars were clustered into 5 clusters with at least 2 per cultivar (Figure 6). The first cluster included the Giza 134, Giza 133, and Giza 136 cultivars. The cultivars Giza 132, Giza 2000, and Giza 128 were discriminated as two neighboring pairs of cultivars. The third cluster consisted of Giza 126 and Giza 137, while Giza 135, Giza 131, Giza 129, and Giza 130 appeared as two neighboring clusters to make up the fourth cluster. The other cultivars—Giza 124, Giza 123, and Giza 125—were located in one group (Figure 6).

**Figure 7:**
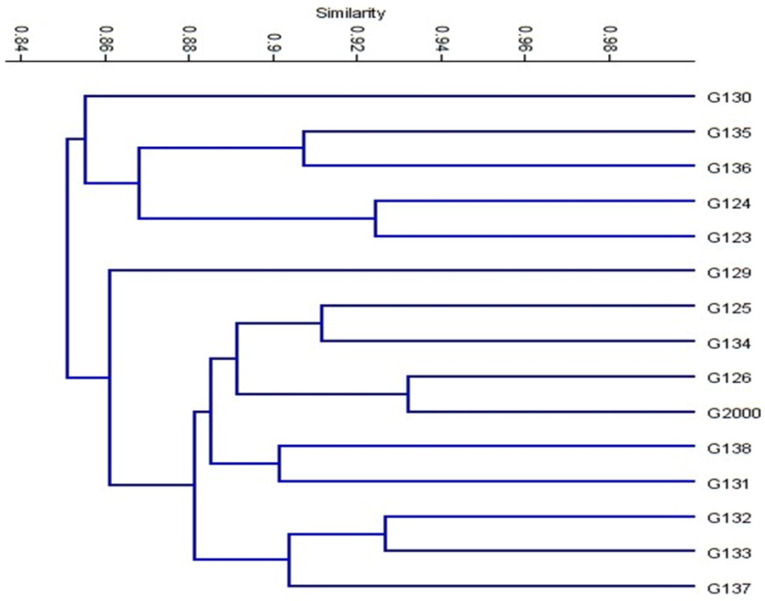
Cluster tree of genetic distance between 15 Egyptian six-rowed barley cultivars, based on the analysis of 15 ISSR primers according to Euclidean distance and the UPGMA algorithm in PAST software.

Based on the ISSR marker data for the studied cultivars, a genetic distance tree was constructed using Dice’s genetic similarity matrix (Figure 7). In this tree, the two pairs (Giza 126 and Giza 2000) and (Giza 132 and Giza 133) were close to the other cultivars. In Egypt, these cultivars are used in human consumption and animal feed. In addition to the malt industry and the beer industry, the ancient Egyptian barley sector dates back to BC. Meanwhile, Giza 132 with Giza 130 and Giza 133 with Giza 130 were less similar to the rest of the barley cultivars, and have been nominated for a crossbreeding program for Egyptian barley breeders. On the other hand, Giza 129 was separated from the rest of the cultivars. All cultivars were distributed in the three clusters. According to the ISSR molecular marker polymorphism, a similarity matrix among the 15 cultivars was derived based on Dice’s coefficient (Table 3). According to the similarity matrix of ISSR analysis, the highest similarity value (93%) was observed between (Giza 133 and Giza 132) and (Giza 2000 and Giza 126). Conversely, the lowest similarity value (80%) was recorded between Giza 130 and (Giza 133 and Giza132), indicating that these cultivars were distantly related, as shown in Table 3 and Figure 7. These distinctive cultivars could be expanded to improve soil properties, reduce fertilizer consumption, increase tolerance to drought and salinity, and facilitate growth in newly reclaimed lands. The results were nearly in agreement with those of previous studies [12,42,43,44].

### 2.4. Biplots

**Figure 8:**
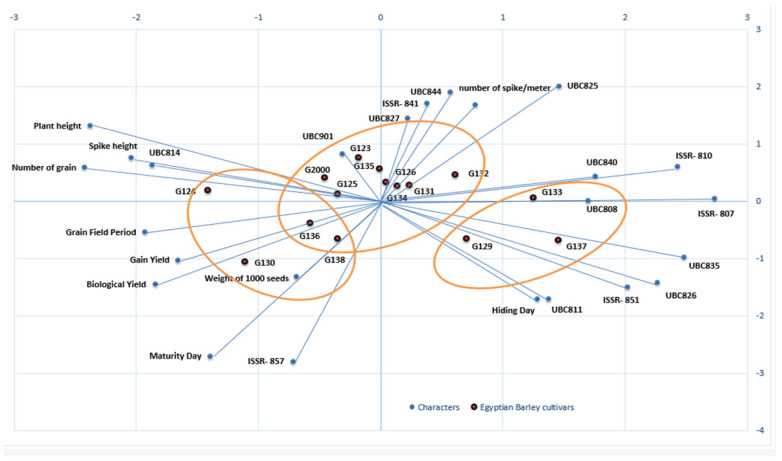
A biplot cluster tree illustrates the genetic distance between 15 Egyptian six-rowed barley cultivars, based on the analysis of 10 morpho-agronomic traits and 15 ISSR primers according to Euclidean distance and the UPGMA algorithm in PAST software.

Biplots were used to reflect the statistical values and their presentation in order to provide supportive information about all of the investigated parameters. Biplots have been used in previous studies to illustrate and present different types of data [45,46,47]. Through the different types of data, the information can be dispersed, but the biplot distributes the genotypes based on all of the traits under study, whether morphological data or molecular data. The biplot in Figure 8 shows the differences between the clusters in the morphological data and the clusters of the molecular data, as well as their interaction; it also clearly demonstrates the effects of each field trait on the genotypes, along with the effects of each initiator molecule on the Egyptian barley genotypes.

To study the interaction between genotype and environment (GE), biplot analysis was utilized [48]. Using the constructed PCA biplot, it became clear which of the 10 morpho-agronomic traits and 15 ISSR primers contributed most to the discrimination of the examined cultivars (Figure 8). The 15 cultivars were divided into 3 groups based on 10 field traits and 15 molecular ISSR primers. The group that included Giza 130, Giza 136, Giza 138, and Giza 126 was the most influenced by the field and morphological characteristics, as shown in Figure 8. This group was established based on maturity day, biological yield, grain yield, weight of 1000 grains, grain field period, and the number of grains. At the same time, the genotypes Giza 129, Giza 137, and Giza 133 were more influenced by the molecular primers associated with age, including ISSRs 807, UBC 835, UBC 826, 851, and UBC 811, as well as hiding day. On the other hand, the third group was affected by plant height characteristics. The number of spikes per square meter, along with the remainder of the molecular parameters, characterized the cultivars Giza 134, Giza 131, Giza 132, Giza 126 Giza 135, Giza 123, Giza 125, and Giza 2000. Generally, when the cultivar falls on the adjective line, it is more impacted by it. Through the current data, we found that the genetic basis of the ISSR molecular markers is dominant over the morphological traits in the first and second groups, while the effect of field traits is predominant in the third and fourth groups, indicating the merging of the field cluster with the molecular cluster into one form in the biplot. Moreover, the contributions of the genes controlling the traits are shown through the molecular parameters, while the environment is shown by the field traits, and the differences in terms of environment and genetics in this study are united by environmental and genetic data [49,50].

### 2.5. DNA Barcoding Loci of mat*K* and rbc*L* Sequencing

DNA barcoding is an essential tool for species identification [51]. Genes from the chloroplast genome—such as *mat*K and *rbc*L—were used for DNA barcoding. The genetic diversity and phylogeny of the studied cultivars were determined by amplification and sequencing of both loci. Four barley cultivars were used for DNA barcoding. Two cultivars, marked by an asterisk (*), had a tough, inedible outer hull around the barley kernel (Giza 123 and Giza 138), while two cultivars marked by two asterisks (**) were characterized by sticks and sprouts that separate from the seed when ripe (Giza 126 and Giza 136) (Table 3). There was 100% amplification success with high specificity of PCR amplification of the *mat*K and *rbc*L regions for all four cultivars, as indicated by sharp DNA bands with no byproducts. The recorded size of the PCR product of the *mat*K region was 900 bp, while for the *rbc*L region it was 600 bp (data not shown). The GenBank accession numbers for *rbc*L in Giza 123, Giza 126, Giza 136, and Giza 138 are MW336986, MW391913, MW336987, and MW391914, respectively. The GenBank accession numbers for *mat*K in Giza 123, Giza 126, Giza 136, and Giza 138 are MW336988, MW336991, MW336990, and MW336989, respectively. To confirm the correct amplification of the *mat*K and *rbc*L sequences, a BLAST function was performed, identifying that all of the sequences were strongly coordinated with *mat*K and *rbc*L of the *Hordeum vulgare* sequences. Sun, et al. [52] assessed the possibility of using five intensively suggested regions (*rbc*L, *mat*K, *trn*H-*psb*A, internal transcribed spacer (*ITS*), and *ITS2*) as DNA barcode candidates to differentiate important species of Brassicaceae in China, in order to establish a new digital identification scheme for economic plants of Brassicaceae. They investigated 58 samples from 27 economic species of Brassicaceae for the success of PCR amplification, intra- and interspecific divergence, DNA barcoding gaps, and identification efficiency. Based on their results, the ITS showed superior discriminative ability, with a rate of 67.2% at the species level when compared with other markers.

Pairwise distances were calculated and evaluated based on the conserved *mat*K and *rbc*L gene sequences, using the WebLogo tool [53]. Additional information on the DNA barcoding regions of *mat*K and *rbc*L in four Egyptian six-rowed cultivars of *Hordeum vulgare* is provided in the Supplementary Materials (Supplementary Figures S1 and S2, respectively); this includes the alignment length, undetermined characters, missing percentages, and variable sites and their proportions, as well as parsimony-informative sites.

**Figure 9:**
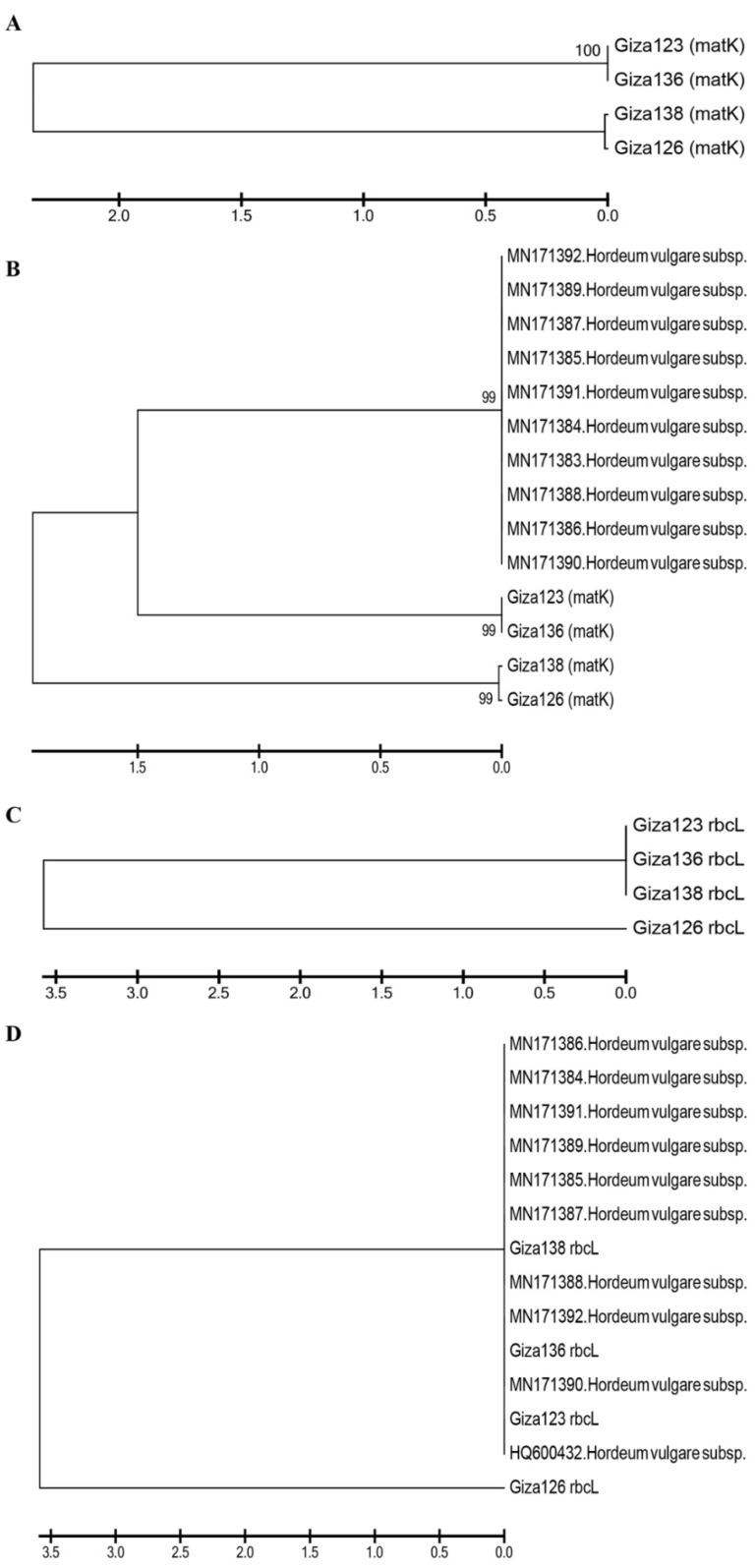
Phylogenetic trees based on (**A**) the *mat*K DNA barcoding region for four six-rowed barley cultivars (Giza 123, Giza 126, Giza 136, and Giza 138); (**B**) the *mat*K DNA barcoding region for four six-rowed barley cultivars, with 10 additional *mat*K sequences of *Hordium vulgaris* L. used as outgroups; (**C**) the *rbc*L DNA barcoding region for four six-rowed barley cultivars; and (**D**) the *rbc*L DNA barcoding region for four six-rowed barley cultivars, with 10 additional *rbc*L sequences of *Hordium vulgaris* L. used as outgroups, using MEGAX software.

Figure 9A illustrates a phylogenetic tree of *mat*K sequence variation using the UPGMA algorithm to discriminate between the four investigated cultivars. To demonstrate the accuracy and efficacy of the created tree, 10 *mat*K sequences were obtained from NCBI and used as outgroups. The tree has three major clusters: the first includes the cultivars in two groups—Giza 123 and Giza 136—and the second includes Giza 126 and Giza 136 (Figure 9B). The third group comprises NCBI outgroup members (Figure 9B). There was a branch length of 1.5, and the bootstrap value displayed next to the branches designates the bootstrap value supporting the node. The Jukes–Cantor method was used to calculate the evolutionary distances based on the base substitutions per site. The bootstrap values were incredibly high (99%), confirming the validity of the tree branching. In the *rbc*L gene region, the four cultivars were distributed into two groups: the first included Giza 123, Giza 136, and Giza 138, while the other contained only Giza 126 (Figure 9C). Additionally, when 10 versions of the identical gene sequences from NCBI were added to GenBank, they resulted in 14 barley genotypes (Figure 9D). The 14 genotypes were distributed into 2 groups: the first group included the cultivar Giza 126 only, while the second group contained the other 13 cultivars (Figure 9D). There was a branch length of 3.5, and the bootstrap value displayed next to the branches designates the bootstrap value supporting the node. The Jukes–Cantor method was used to calculate the evolutionary distances based on the base substitutions per site. The ambiguous plant pairwise deletion option was used. In addition, the rest of the Egyptian cultivars were compared with GenBank’s publications, where it was noted that the closest to Giza 123 were the HQ800432 and MN171390 versions. Using DNA barcoding, species could be classified quickly without relying on morphological characteristics. This technique uses DNA fragments of relatively small size as tags to describe or discover species [54].

On the other hand, the MN171392 version was close to Giza 136. Moreover, Giza 138 fell between two versions MN171388 and MN171387 (Figure 9D). After adding NCBI GenBank accession numbers, sequences had 24 genotypes for each region of the *rbc*L and *mat*K genes. The distribution of the GenBank NCBI accession numbers and the Egyptian cultivars did not differ from that of each gene separately from the regions of the *rbc*L and *mat*K genes; however, the similarity percentage was as follows in the *mat*K gene: The GenBank accession numbers of *rbc*L and *mat*K in Giza 123 and Giza 136 were distributed at 99% similarity, whereas the similarity rate of Giza 138 and Giza 126 reached 66% in the area of the genome. Meanwhile, in the *rbc*L gene region, the similarity rate was 56% for Giza 126, while the rest of the GenBank accession numbers and Egyptian cultivars were distributed at a similarity rate of 99%.

The results of the current study show that *rbc*L is less mutable than *mat*K in terms of sequence variability among the examined cultivars. Previous studies used the *mat*K region in many phylogenetic analyses of flowering plants, due to its conservative mode of evolution [55,56]. Four cultivars were differentiated in the present study according to *mat*K sequence variation, using 10 outgroup sequences from NCBI (Figure 9). The phylogenetic tree created using 10 NCBI-extracted *mat*K sequences of *Hordeum vulgare* confirmed the outstanding finding of separating the four cultivars Giza 123, Giza 126, Giza 136, and Giza 138, along with the *Hordeum vulgare* NCBI *mat*K sequence and its subspecies. Nevertheless, Giza 123 and Giza 136 were separated with the *Hordeum vulgare* NCBI *mat*K sequence of the NCBI accession numbers, suggesting sequence homology. In the second cluster, Giza 138 and Giza 126-super-supreme were in the same group, and shared high homology in *mat*K sequences. The *rbc*L region was used to distinguish between wild parents, as well as being used as precise sequences to distinguish between different degrees of biological diversity [57,58,59]. In addition to providing potentially helpful information for genome-assisted research, the present study also provides useful information for crop improvement.

## 3. Materials and Methods

### 3.1. Plant Materials

This study examined 15 Egyptian barley cultivars (all six-rowed). Those cultivars were selected because they are more critical to the Egyptian barley industry than the two-rowed lines. Viable grains of the studied cultivars were obtained from the Barley Research Department (BRD), Field Crop Research Institute (FCRI), Agricultural Research Center (ARC), Giza, Egypt, during two seasons: 2018/2019 and 2019/2020 (Table 4). These cultivars were chosen based on the recommendations of the barley breeders and the beer industry for their salinity and drought tolerance, high yield, and phytochemical characteristics—such as mineral elements and malt content.

### 3.2. Morphological Traits and Experimental Design

Two field experiments were carried out at El-Giza Agricultural Research Station (Giza, Egypt) during the successive winter seasons of 2018/2019 and 2019/2020 to study the morphological traits of the different cultivars. To differentiate between the studied cultivars based on morphological characteristics, the following parameters were recorded: days to 50% heading (HD), days to 50% maturity (MD), grain filling period (GFP) (days), plant height (PH) (cm), spike length (SL) (cm), number of grains per spike (average of 10 spikes per square meter), number of spikes per m^2^ (No. Sp./m^2^), weight of 1000 grains (g), biological yield (BY) (t/ha), and grain yield (GY) (Kg/ha). The grain filling period (GFP) was calculated using the following formula:

Grain filing period=maturity days−flowering day

A randomized complete block design (RCBD) with four replications was used. The plot size was 4 rows that were each 3 m long and 20 cm apart. Analysis of variance and least significant difference (LSD) at 5% were used for comparison between the cultivars.

### 3.3. Scanning Electron Microscopy (SEM)

Four barley cultivars (Giza 123, Giza 126, Giza 136, and Giza 138) were studied using SEM. Those four cultivars were chosen based on the recommendations of the plant breeders. The chosen cultivars have high production demand and can withstand harsh conditions; they also have excellent synthetic qualities, which is the reason for their examination. For example, Giza 123 tolerates harsh conditions and high salinity levels; Giza 126 has excellent drought tolerance, and is grown under the rain on the northern coast of Egypt, while Giza 136 and Giza 138 are characterized by high yield under all conditions. Viable grains of the studied cultivars were obtained during the season of 2019. The clean and dry seed samples of the studied barley cultivars were placed on double-stick tape mounted on a copper electron microscope holder. The specimens were coated with gold, and then investigated and photographed with a JEOL JSM T200 at 25 kV, in the electron microscope unit of Mansoura University, Mansoura, Egypt. Seed coat technical terms were based on the works of Koul, et al. [71],Murley [72],and Stearn [73].

### 3.4. ISSR Molecular Markers

#### 3.4.1. Extraction of Genomic DNA

Fresh leaf tissue (0.1 g of combined samples from three different plants) ground in liquid nitrogen with a mortar and pestle was used to extract genomic DNA using the cetyl trimethyl ammonium bromide (CTAB) protocol [74]. DNA concentration and purity for all samples were determined spectroscopically at 260 and 280 nm, respectively. DNA samples were stored at −20 °C for subsequent molecular analysis.

#### 3.4.2. ISSR Amplification

ISSR amplification reactions were carried out in equal volumes (15 µL) containing 7.5 µL of 2× Master Mix (OnePCR^TM^, GeneDireX, Inc., Taipei, Taiwan), 1 µL of DNA template (10 ng/µL), and 1 μL of primer. The names and sequences of the ISSR primers used in the current study are listed in Table 2. The amplification reaction was performed using a T100^TM^ Thermal Cycler (Bio-Rad^®^ Laboratories, Hercules, CA, USA). The polymerase chain reaction (PCR) program was as follows: initial denaturation at 94 °C for 4 min, followed by 30 cycles, with the first step at 94 °C for 30 s (denaturation), the second step varying between 46 and 52 °C—depending on the GC content of each primer—for 45 s (annealing), and the third step (extension) at 72 °C for 1 min, followed by a final extension step at 72 °C for 7 min. The reaction was stopped by maintaining the tubes at 4 °C for at least 30 min. Amplification products were separated via electrophoresis on 1.5% agarose gel in 1× TBE buffer (Tris-borate-EDTA). The gels were stained with 0.5 µg mL^−1^ ethidium bromide (EtBr) solution (Thermo Fisher Scientific, Carlsbad, CA, USA). Then, the gel was documented using a Bio-Rad ChemiDoc^TM^ MP gel documentation system (Bio-Rad). The primers that gave reproducible results were used for data analysis. Polymorphism indices were calculated using iMEC (Online Marker Efficiency Calculator) (https://irscope.shinyapps.io/iMEC/) [75]. ClustVis, a web tool for visualizing clustering of multivariate data, was used to construct heatmaps (https://biit.cs.ut.ee/clustvis/) [41].

### 3.5. DNA Barcoding of Plastid Genes rbc*L* and mat*K*

DNA barcoding of sequences for the *rbc*L and *mat*K genes was performed using computational analysis. BioEdit software version 7.2.5 (https://bioedit.software.informer.com) was used to analyze and assemble the *rbc*L and *mat*K gene sequences for every cultivar. Using the BLAST function (https://www.ncbi.nlm.nih.gov), the sequences were compared with all accessible sequences in the database. The primers used for barcoding of the *rbc*L and *mat*K genes are listed in Table 5. The PCR program to amplify the two genes was as follows: initial denaturation at 94 °C for 4 min, followed by 40 cycles, with a denaturation step at 94 °C for 30 s, annealing step at 45 °C for 30 s, and elongation step at 72 °C for 30 s, followed by a final extension step at 72 °C for 7 min, after which it was maintained at 4 °C to stop the reaction. The PCR products were subsequently electrophoresed on 1.5% *w/v* agarose, stained with 0.5 µg mL^−1^ EtBr solution (Thermo Fisher Scientific) in 1× TBE buffer, and visualized as described for the ISSR PCR amplification. The PCR products of the *mat*K and *rbc*L genes were recovered from agarose gel and purified using the Monarch DNA Gel Extraction Kit (New England Biolabs, Inc., Ipswich, MA, USA), according to the manufacturer’s instructions. The purified *mat*K and *rbc*L amplicons were cloned into pGEM^®^-T Easy Vector Systems (Promega Corporation, Madison, WI, USA) before sequencing. After being transformed into the competent cells of the *E. coli* strain DH5α (Promega, Madison, WI, USA), the positive recombinants were identified via anti-ampicillin selection and verified by PCR screening. Three of the positive clones were sequenced using the ABI PRISM Big Dye Terminator Cycle Sequencing Ready Reaction Kit (Applied Biosystems, Waltham, MA, USA) in conjunction with ABI PRISM (3100 Genetic Analyzer, Macrogen DNA Sequencing Services, Seoul, Korea), as described by Badr, et al. [76]. Using Gblocks software version 0.91b, the revealed nucleotide sequence was assembled [77,78].

Online ClustalW2 software (https://www.ebi.ac.uk/Tools/msa/clustalw2/) was used to align multiple nucleotide sequences, which were double-checked using MEGAX (www.megasoftware.net). Gblocks version 0.91b [77,78] was used to review and assess the gaps in the positions. MEGAX software using the UPGMA algorithm was used to perform the phylogenetic analysis. Confidence of the clustering was attained using SEQBOOT (https://csbf.stanford.edu/phylip/seqboot.html). The sequence logos of the multiple sequence alignments were generated using the WebLogo tool [53]. Additionally, a principal component analysis (PCA) biplot based on the morpho-agronomic data matrix was constructed via multivariate analysis using PAST software versiong 4.02 (https://www.nhm.uio.no/english/research/infrastructure/past/).

### 3.6. Statistical Analysis

Standard analysis of variance (ANOVA) using least significant differences (LSD) was utilized to estimate the significant differences between the 15 cultivars of six-rowed barley [81]. Dendrogram cluster analysis was used to arrange a set of variables into clusters. A cluster analysis was performed using Euclidean distance and similarity levels [82,83]. ISSR markers that generated clear, distinct, and reproducible bands were recorded as (0) for absence or (1) for presence. The ability of ISSR primers to differentiate between investigated genotypes was analyzed by calculating the polymorphic information content (PIC) [84]. Resolving power (Rp) was measured following the formula of Gilbert, et al. [85]. Additionally, marker index (MI) and effective multiplex ratio (EMR) values were calculated. For the calculation of the coefficient of genetic similarity matrix, and for the construction of a distance tree illustrating the relationships between the tested genotypes, the ISSR marker matrices were used in combination with the unweighted pair group method with arithmetic mean (UPGMA) in PAST software version 4.02 [86]. Furthermore, by using PAST software version 4.02 [86], a PCA scatter diagram was constructed based on a Dice coefficient genetic similarity matrix. ClustVis, a web tool for visualizing clustering of multivariate data, was used to constructe heatmaps (https://biit.cs.ut.ee/clustvis/) [41].

## 4. Conclusions

Barley plays a vital role in Egypt in terms of agricultural development and added value, as it is used in new and marginal lands. Today, there is expansion in its cultivation due to its high adaptation to water scarcity and other harsh conditions. Thus, barley is added to wheat flour to increase the nutritional value of bread, and is also used in the manufacture of beer and malt. These cultivars are the most common forms of barley in Egypt, so they have been studied for their economic importance in terms of added value and sustainable development. Despite the differences at the molecular level, the examined Egyptian cultivars reflected similarities in terms of field performance under the optimal environment, exhibiting no differences in terms of field characteristics. These cultivars were closely distributed in a genetic tree, similar to the genetic tree based on the molecular description. These differences enable the breeders to choose the best of these cultivars from the most divergent, and to exclude the least different. Moreover, the electron microscope examination reflected differences in the seed surface characteristic, which helps in understanding the chemical content of the Egyptian barley grains and their economic importance. Interestingly, the sequencing results of four cultivars showed that the *rbc*L gene referred to the uniqueness of these four cultivars compared to the sequence database. Nevertheless, the second gene *mat*K revealed that these cultivars are very similar to the GenBank accession numbers. Additionally, the production of new sequences was added to the molecular information about the Egyptian barley cultivars, showing the differences between the Egyptian and European cultivars—especially since Egypt is one of barley’s countries of origin. These results will potentially enhance breeding programs and aid in the development of new adaptive or high-yield barley cultivars with specific improved traits.

## Figures and Tables

**Table 1:** The seed coat characteristics of four Egyptian six-rowed barley (*Hordeum vulgare* L.) cultivars (Giza 123, Giza 126, Giza 136, and Giza 138).

	Giza 138	Giza 136	Giza 126	Giza 123
Frequency pattern in 100 µm^2^	12.17 ± 1.69	4.83 ± 0.52	4.67 ± 0.51	8.17 ± 0.99
The elevation folding of rugose (µm)	12.67 ± 2.04	12.67 ± 2.04	11.00 ± 1.73	14.67 ± 2.43
The extent of the rugose surface (length, µm)	18.00 ± 3.01	18.67 ± 3.13	16.00 ± 2.61	14.00 ± 2.27

**Table 2:** ISSR marker profiles for 15 Egyptian six-rowed barley cultivars.

Primer No.	Name	Sequence	MB	POU	UB	PWU	TNB	P%	MBF	PIC	RP	EMR	MI
1	UBC 825	(AC)_7_ T	7	2	0	2	9	22	1.0	0.32	5.27	8.87	0.02
2	UBC 835	(Ag)_8_ YC	7	7	0	7	14	50	0.8	0.26	5.86	12.07	0.02
3	UBC 814	(CT)_7_ CAT	2	3	1	4	6	67	0.6	0.37	12.67	8.33	0.05
4	UBC 826	(AC)_8_ C	6	4	0	4	10	40	0.8	0.30	7.40	11.30	0.03
5	UBC 827	(AC)_8_ G	6	5	1	6	12	50	0.6	0.36	11.17	9.42	0.02
6	UBC 840	(gA)_8_ TT	2	6	0	6	8	75	0.6	0.37	10.50	8.75	0.04
7	UBC 808	(Ag)_8_ C	4	3	0	3	7	43	0.7	0.35	10.00	10.00	0.04
8	UBC 811	(gA)_7_ gC	5	3	0	3	8	38	0.7	0.32	8.25	10.88	0.04
9	UBC 844A	(CT)_8_ AC	1	4	0	4	5	80	0.4	0.37	10.40	6.20	0.04
10	UBC 901	(CA)_8_ RY	3	2	0	2	5	40	0.8	0.27	6.00	12.00	0.05
11	807	(AG)_8_ T	5	4	1	5	10	50	0.7	0.35	10.00	10.00	0.03
12	810	(GA)_8_ T	4	6	0	6	10	60	0.7	0.33	8.60	10.70	0.03
13	841	(GA)_8_ YC	3	4	0	4	7	57	0.7	0.33	9.14	10.43	0.04
14	857	(AC)_8_ YG	5	2	0	2	7	29	0.8	0.28	6.29	11.86	0.04
15	851	(GT)_8_ YG	4	3	1	4	8	50	0.6	0.36	11.25	9.38	0.04
Total			64	58	4	62	126	-		-	-	-	-
Mean			4.27	3.87	0.27	4.13	8.40	50.07		49	0.31	0.31	8.85

Each estimated parameter’s minimum and maximum values are highlighted in yellow. MB: monomorphic bands; POU: polymorphic without uniqueness; UB: unique bands; PWU: polymorphic with uniqueness; TNB: total number of bands; P%: polymorphism (%); MBF: mean of band frequency; PIC: polymorphism information content; RP: resolving power; EMR: effective multiplex ratio; MI: marker index.

**Table 3:** Genetic similarity of the 15 Egyptian six-rowed barley cultivars, based on ISSR fingerprinting.

	G123	G124	G125	G126	G2000	G132	G133	G134	G137	G138	G129	G130	G131	G135	G136
G123	100														
G124	92	100													
G125	90	90	100												
G126	90	86	91	100											
G2000	87	88	91	93	100										
G132	86	86	91	91	89	100									
G133	84	83	89	87	86	93	100								
G134	86	87	91	86	88	87	91	100							
G137	83	83	87	86	84	91	90	86	100						
G138	84	84	90	90	89	87	88	89	89	100					
G129	80	82	85	82	82	86	88	89	86	87	100				
G130	84	86	84	84	84	80	80	83	82	89	83	100			
G131	86	88	89	89	86	90	87	86	89	90	88	88	100		
G135	87	85	83	86	86	82	85	86	82	85	83	86	88	100	
G136	87	88	87	88	87	84	87	87	81	87	86	86	86	91	100

**Table 4:** Name, origin, and year of release of the Egyptian six-rowed barley cultivars as recorded by the Barley Research Department, Field Crops Research Institute, Agricultural Research Center, Egypt, and the GenBank accession numbers for the *mat*K and *rbc*L genes for four six-rowed barley cultivars (Giza 123, Giza 126, Giza 136, and Giza 138).

No.	Cultivar	Origin (Year of Release)	*rbc*L GenBank	*mat*K GenBank	Kind	Pedigree	References
1	Giza 123	Egypt (1998)	MW336986	MW336988	Naked *	Giza117/FAO86	[60]
2	Giza 124	Egypt (1998)	NA	NA	Naked	Giza 117/Bahtim 52//Giza 118/ FAO86	[61]
3	Giza 125	Egypt (1995)	NA	NA	Naked	Giza 117/Bahtim52//Giza 118/ FAO86(2)	[62]
4	Giza 126	Egypt (1995)	MW391913	MW336991	Naked	Baladi Bahteem/S D729-Por12762-BC	[62]
5	Giza 129	Egypt (2003)	NA	NA	Hull-less **	Deir Alla106/Cel//As 46/Aths *2	[63]
6	Giza 2000	Egypt (2003)	NA	NA	Naked	Giza117/Bahteem52//Giza118/FAO86/3/Baladi16/Gem	[64]
7	Giza 130	Egypt (2003)	NA	NA	Hull-less	Comp Cross 229//Bco.Mr./DZ02391/3/Deir Alla 106	[63]
8	Giza 131	Egypt (2003)	NA	NA	Hull-less	CM67-B/CENTENO/CAM-B/ROW 906.73/4/GLORIA-BAR-COME-B/5/FALCON-BAR/6/LINO	[65]
9	Giza 132	Egypt (2006)	NA	NA	Naked	Rihane-05//As 46/Aths *2 Aths/Lignee 686	[66]
10	Giza 133	Egypt (2018)	NA	NA	Naked	Carbo/Gustoe	[67,68]
11	Giza 134	Egypt (2019)	NA	NA	Naked	Alanda-01/4/WI2291/3/Api/CM67//L2966-69	[67,68]
12	Giza 135	Egypt (2019)	NA	NA	Hull-less	ZARZA/BERMEJO/4/DS4931//GLORIA-BAR/COPAL/3/SEN/5/AYAROSA	[69]
13	Giza 136	Egypt (2019)	MW336987	MW336990	Hull-less **	PLAISANT/7/CLN-B/4/S.P-B/LIGNEE640/3/S.P-B/GLORIA-BAR/COME-B/5/FALCON-BAR/6/LINO	[70]
14	Giza 137	Egypt (2019)	NA	NA	Naked	Giza 118/4/Rhn-03/3/Mr25-//Att//Mari/Aths *3-02	[32]
15	Giza 138	Egypt (2019)	MW391914	MW336989	Naked *	Acsad1164/3/Mari/Aths *2//M-Att-73-337-1/5/Aths/lignee686/3/Deir Alla106//Sv.Asa/Attiki/4/Cen/Bglo.”S”	[32]

NA: not available; *: has a tough, inedible outer hull around the barley kernel; **: hull-less—the sticks and sprouts separate from the bean when ripe, and the chromosome number is 2n = 2× = 14 (map view: “barley genome at ncbi.nlm.nih.gov”; retrieved 6 October 2014).

**Table 5:** Primer names, sequences, and product sizes for the *rbc*L and *mat*K genes’ DNA barcoding.

Primer Names	Sequence	Product Size	References
*rbc*L-F	5’-ATGTCACCACAAACAGAGACTAAAGC-3’	600 bp	[79]
*rbc*L-*R*	5’-TCGCATGTACCTGCAGTAGC-3’		
*mat*K-*F*	5’-CGATCTATTCATTCAATATTTC-3’	900 bp	[80]
*mat*K-*R*	5’-TCTAGCACACGAAAGTCGAAGT-3’		

## Data Availability

All the obtained data presented in this the article or supplementary material.

## Supplementary Materials

The following are available online at https://www.mdpi.com/article/10.3390/plants10112527/s1: Figure S1: Sequence logos of the multiple sequence alignment of *mat*K; Figure S2: Sequence logos of the multiple sequence alignment of *rbc*L.
