# Supplementary figures and images for: Morphological and Molecular Characterization of Some Egyptian Six-Rowed Barley (Hordeum vulgare L.)
Source: Plants (Basel). 2021 Nov 20;10(11):2527. doi: 10.3390/plants10112527 (PMC8619447; doi:10.3390/plants10112527)

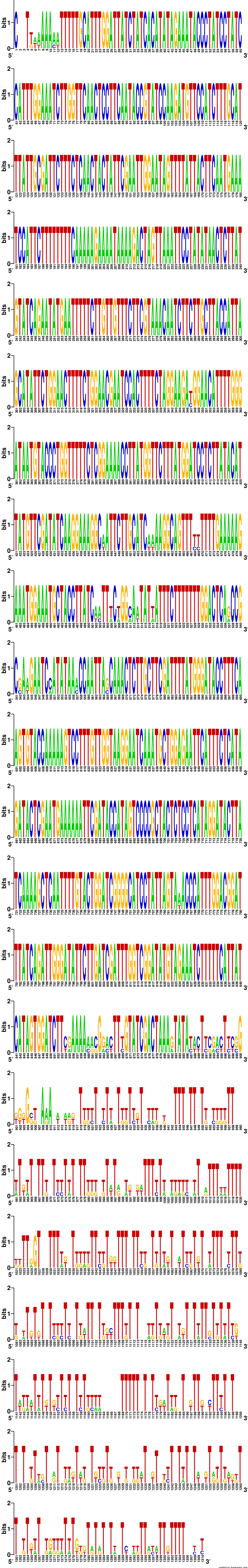

Supplement: Supplementary file 1 [file plants-10-02527-s001.zip › Figure S1. sequence logos of the multiple sequences alignment of matK.pdf]

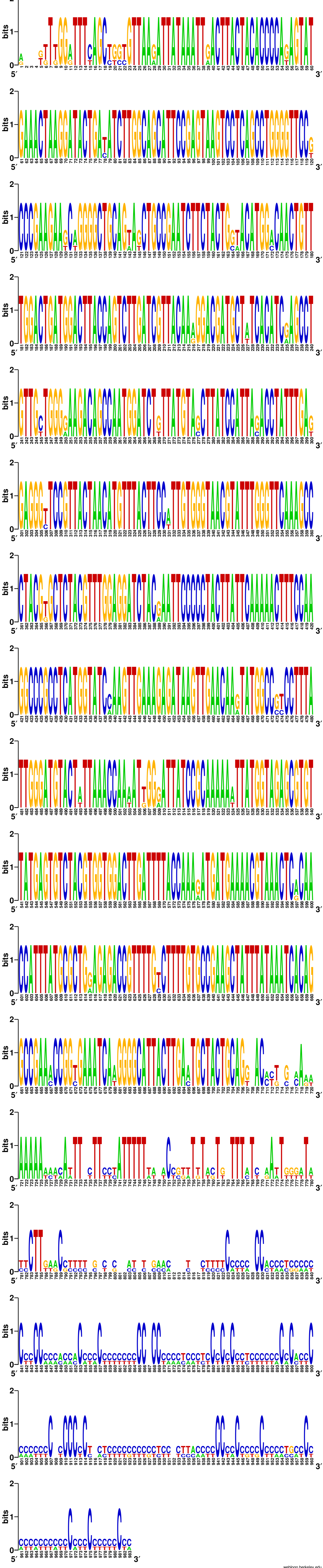

Supplement: Supplementary file 1 [file plants-10-02527-s001.zip › Figure S2. sequence logos of the multiple sequences alignment of rbcL.pdf]
